# The Efficacy and Safety of Endoscopic Ultrasound-Guided Celiac Plexus Neurolysis for Treatment of Pain in Patients with Pancreatic Cancer

**DOI:** 10.1155/2012/503098

**Published:** 2012-02-07

**Authors:** Anna Wiechowska-Kozłowska, Klaudiusz Boer, Maciej Wójcicki, Piotr Milkiewicz

**Affiliations:** ^1^Department of Endoscopy, Ministry of Internal Affairs Hospital, Ul. Jagielonska 44, 70-362 Szczecin, Poland; ^2^Department of Hepato-Pancreato-Biliary Surgery and Liver Transplantation, M. Curie Hospital, 71-455 Szczecin, Poland; ^3^The Liver Unit, Pomeranian Medical University, 70-111 Szczecin, Poland

## Abstract

*Introduction.* Celiac plexus neurolysis is used in pain management of patients with advanced and unresectable pancreatic cancer. We retrospectively analyzed efficacy and safety of endoscopic ultrasound- (EUS-) guided celiac plexus neurolysis in patients treated in our unit. *Methods.* Twenty nine subjects with unresectable pancreatic cancer and severe pain despite pharmacological treatment underwent EUS-guided celiac plexus neurolysis with 98% ethanol. Patients scored their pain according to a 0–10 point scale and were interviewed 1-2 weeks and 2-3 months after the procedure. *Results.* Twenty five (86%) patients reported improvement in their pain at 1-2 weeks following the procedure. Of these, 7 (24%) reported substantial improvement (decrease in pain by more than 50%) and 4 (14%) complete disappearance of pain. Pain relief was still present in 76% of patients after 2-3 months. Treatment-related side effects included hypotonia in 1 patient, severe pain immediately postprocedure in 2 patients, and short episodes of diarrhea in 3 patients. *Conclusion.* Endoscopic ultrasound- (EUS-) guided celiac plexus neurolysis is a safe and effective treatment of severe pain from advanced pancreatic cancer.

## 1. Introduction

Treatment of pain in patients with advanced pancreatic cancer is one of the most important goals of palliative care. It is estimated that pain occurs in 80–85% of patients with unresectable pancreatic tumors [1, 2]. Despite the improved effectiveness of pharmacotherapy, treatment of severe pain from inoperable pancreatic cancer remains an important clinical issue. Conventional drugs do not provide adequate analgesia and many adverse effects are also seen with opioids. Therefore, interventional or surgical methods of pain treatment are attractive alternatives in such patients [3, 4]. For example, celiac plexus neurolysis destroys the plexus that plays a crucial role in transmitting pain of pancreatic origin. The procedure involves direct injection of a chemical agent, a solution of alcohol or glycol, into the celiac plexus ganglia [1–5].

 Percutaneous celiac plexus neurolysis was first performed by Kapisa in 1914. Since then, it has been performed by many techniques for access, and with a variety of chemicals [6]. Percutaneous neurolysis under radiologic guidance is the most commonly applied. The needle is first introduced into the region of the celiac plexus under fluoroscopic guidance. A mixture of alcohol or phenol with the addition of contrast medium is administered. A limitation of this method is the lack of direct visualization of the celiac trunk, resulting in only an approximation of location of the puncture site. As a result, the risk of vascular or neurologic complications is higher when accessed from the lumbar region. CT-guided neurolysis is a modification with similar limitations as fluoroscopy [4].

Intraoperative celiac plexus neurolysis during surgery is seldomly used because in most cases, the diagnosis of unresectable pancreatic cancer is established without the need for laparotomy. Such patients usually require endoscopic stenting of the biliary tree and adequate pain control [3].

 Endoscopic ultrasound- (EUS-) guided celiac plexus neurolysis was first described by Wiersema in 1996 [7]. The authors visualized the celiac plexus with EUS and then performed neurolysis via the transgastric route, achieving results comparable to percutaneous neurolysis. In the following 10 years, the endoscopic technique has been accepted as an alternative method of celiac plexus neurolysis, and is now applied in many centers [8–21].

We aimed to assess the safety and efficacy of EUS-guided celiac plexus neurolysis for pain management in patients with advanced and unresectable pancreatic cancer. This is the first reported experience with this technique in Poland.

## 2. Methods

Thirty two patients diagnosed with advanced and unresectable pancreatic adenocarcinoma were selected as candidates for EUS-guided celiac plexus neurolysis. The indication in all cases was severe abdominal pain requiring the use of opioids. Neurolysis was not performed in 3 patients because of the inability to visualize the celiac plexus with EUS due to atypical anatomy. Thus, between May 2008 and May 2009, 29 patients ultimately underwent EUS-guided celiac plexus neurolysis. All procedures were performed by 2 gastroenterologists (A. Wiechowska-Kozłowska and P. Milkiewicz) with extensive experience in EUS (both performed more than 1000 examinations). The linear type EUS endoscope (Olympus GF-UCT 160-OL5) with “spray” needles (ECHO 20 CPN, Cook Ireland) was used in all cases.

Fourteen (48%) men and 15 (52%) women, mean age 62 (range 33–81) years, underwent the procedure. The diagnosis of advanced and unresectable cancer of the pancreatic head (*n* = 13; 45%), the head and body (*n* = 3, 10%), the body (*n* = 9; 31%), and the tail of the pancreas (*n* = 4; 14%) was made based on abdominal computed tomography and EUS. Tumors were considered unresectable when distant metastases and/or locally advanced tumors were present (i.e., tumor infiltration of the celiac trunk, superior mesenteric artery or vein, and the retroperitoneum and periaortic area). Eighteen (56%) patients had advanced local disease and 14 (44%) were diagnosed with metastasis disease. Adenocarcinoma of the pancreas was confirmed in all patients with fine-needle aspiration biopsy under percutaneous (*n* = 11) or endoscopic (*n* = 18) ultrasound guidance. 11 (38%) patients underwent palliative chemotherapy with gemcitabine.

Contraindications to the procedure included coagulation disorders (INR > 1.5), platelet count <50,000, or previous disease and treatment of the upper gastrointestinal tract that would make endoscopic access impossible. These contraindications were not present in included patients. After discussing the principles of the procedure, its consequences, the possibility of partial or no reduction of pain, and complications, informed consent was obtained in all patients.

**Figure 1:**
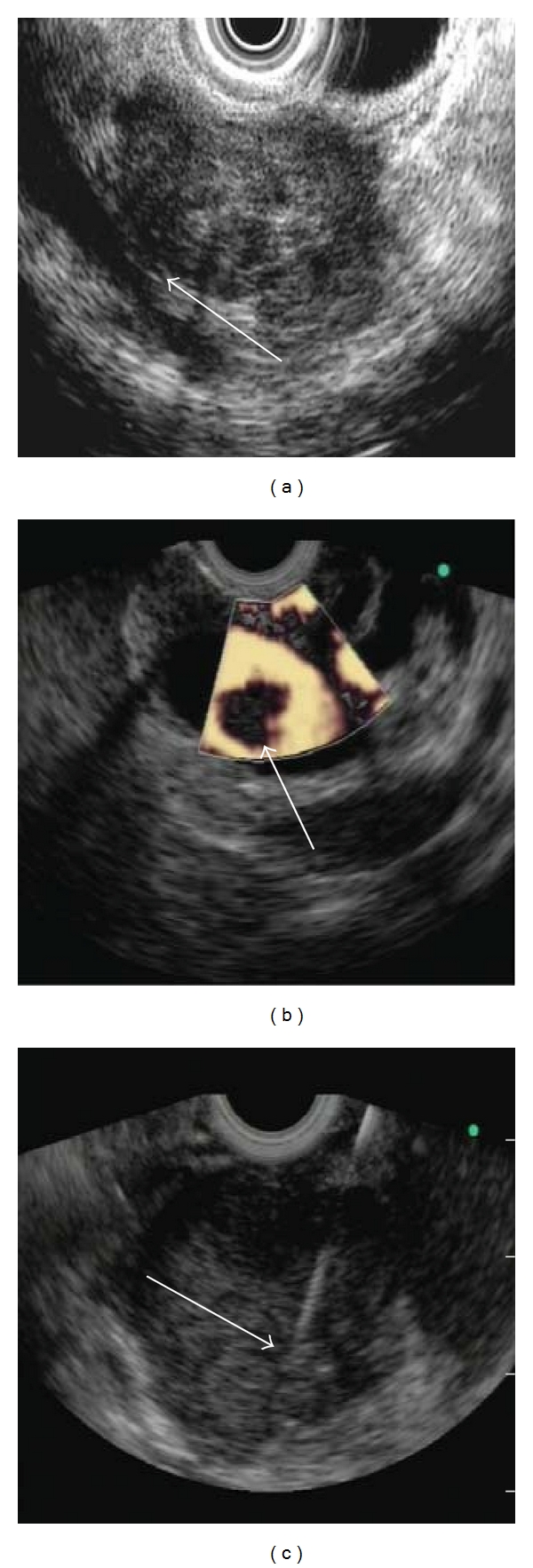
Images of endoscopic ultrasound in advanced pancreatic cancer: (a) tumor invading the vasculature, (b) portal vein thrombosis in advanced pancreatic cancer, (c) fine-needle aspiration biopsy of the pancreatic tumor.

**Figure 2:**
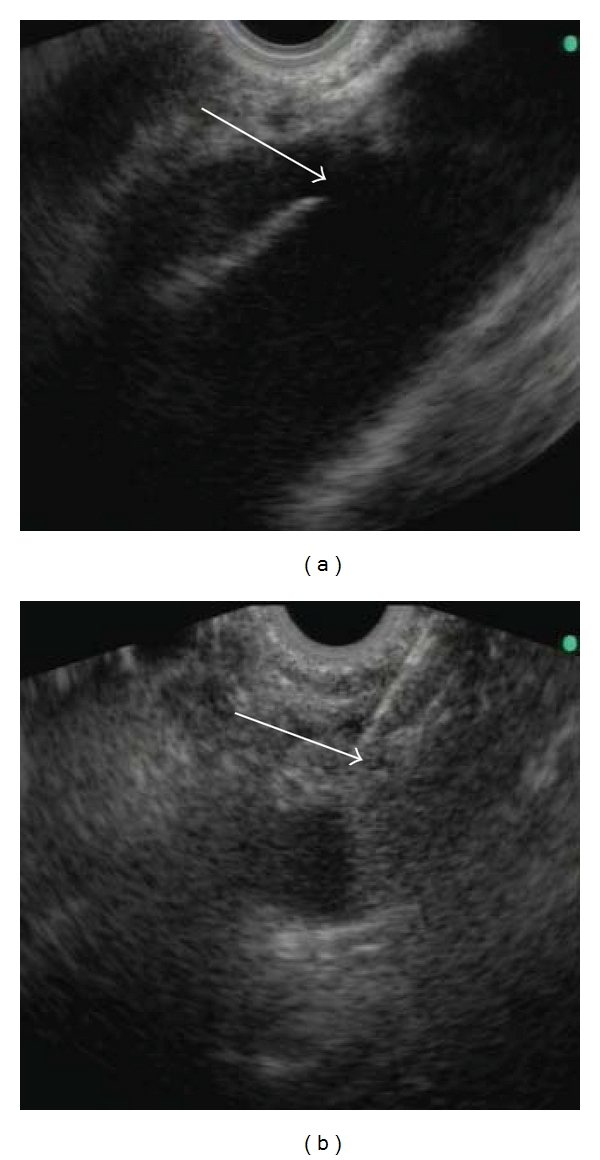
Endoscopic ultrasound-guided celiac plexus neurolysis: (a) typical location of the plexus with the celiac trunk (arrow) at the aorta, (b) puncture of the celiac plexus with administration of alcohol under endoscopic ultrasound guidance.

 The procedure was performed in the left lateral position after intravenous administration of 2.5 mg of midazolam. Clinical parameters such as heart rate, blood pressure, oxygen saturation, and ECG were routinely monitored during the procedure. The antibiotics prophylactic was not used. The first stage was assessment of tumor location, confirming its advanced and unresectable stage (Figure 1). Anatomic landmarks (celiac trunk and aorta visible from the lesser curvature of the stomach) were visualized first. The needle was introduced directly into the celiac plexus and surrounding area under direct visualization of the vessels with the Doppler mode. The aspiration test was routinely performed (2 mL of saline followed by aspiration), in order to exclude intravascular puncture. A small amount of analgesic (2 mL of 2% lidocaine) was administered, followed by injection of 98% alcohol solution (Figure 2). This was performed three times: twice on either side of the aorta and 1 directy to the celiac plexus. Altogether, 3 punctures of the celiac plexus were performed with the total application of 6 mL of lidocaine and 20 mL of 98% alcohol. During alcohol injection, a typical hyperechogenic shadow was observed and patients experienced exacerbation of pain in this region despite administration of analgesia.

After the procedure, patients were observed for 24 hours, with clinical evaluation and measurement of vital signs. 27 patients were discharged on the next day while 2 patients remained in the hospital for 2 days due to exacerbating pain. All the patients were instructed to attempt to gradually discontinue the use of pain medication. The assessment of efficacy and related morbidity was based upon a survey carried out prior to the procedure; on day 1, 1-2 weeks, and 2-3 months following the procedure. The effectiveness of treatment was assessed based on the 11-point pain scale (0 points, no pain; 10 points, maximal pain). The reduction or discontinuation of pain medication was also considered. The incidence and types of complications were evaluated by clinical evaluation during and after the procedure, and by surveying all patients on the degree of pain, changes in bowel movements, neurological disturbances, and other clinical symptoms. Analysis was retrospective and was based on the hospital and endoscopy suit charts.

## 3. Results

**Figure 3:**
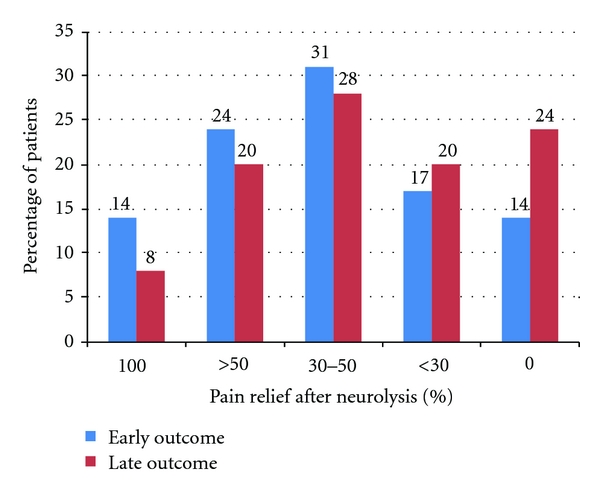
Efficacy of endoscopic ultrasound-guided celiac plexus neurolysis: (i) early outcome (1-2 weeks after treatment), (ii) late outcome (2-3 months after treatment).

An average pain score of 7.9 (range 6–10) was observed in all patients prior to the procedure, requiring the use of nonsteroid anti-inflammatory drugs and narcotic analgesics. One-two weeks following treatment, full pain resolution (0-1 points) was observed in 4 (14%) patients, who completely stopped taking pain medications. Seven (24%) patients had a reduction in pain by more than 50% while 9 (31%) patients had a reduction in pain by 30–50%. In 5 (17%) patients, a small improvement (reduction of pain by <30%) was found. In 4 (14%) patients, pain remained unchanged. Two-three months following the procedure, 4 patients died due to disease progression. These included 1 patient in whom neurolysis was fully effective, 1 patient with pain reduction by 30–50%, 1 patient with pain reduction of <30%, and 1 in whom the procedure was ineffective. Subsequent assessment (2-3 months postprocedure) was performed in 25 patients. Two (8%) patients were pain free and 5 (20%) patients maintained pain relief of more than 50%. Seven (28%) patients reported a 30–50% pain reduction while 5 (20%) and 6 (24%) patients had slight (<30%) or no improvement, respectively (Figure 3).

A short but significant episode of hypotension requiring intervention occurred in 1 patient immediately after procedure. This normalized after treatment with an i.v. saline. Two patients reported a temporary but significant increase in pain immediately after procedure, requiring analgesics in increasing doses during the hospital stay. Both patients were discharged home after two days. One patient was pain free at discharge and 1 had a significant (>50%) reduction in pain. Three patients reported an increased frequency of bowel movements (4-5 stools daily) although no chronic diarrhea was observed in any patient.

## 4. Discussion

Celiac plexus neurolysis for pain management has been used for almost 100 years in patients with advanced abdominal malignancy [6]. The procedure is performed either percutaneously or intraoperatively, with varying efficacy. According to the metaanalysis of 24 studies, including 1145 patients who underwent the percutaneous technique (mostly from the posterior approach), pain reduction was observed in 90% and 70–90% of patients at 2 weeks and 3 months following the procedure, respectively [4]. Patients who underwent percutaneous neurolysis experienced significant pain relief, enabling reduction of analgesic doses and improved quality of life [2, 14–16, 20]. However, serious neurological complications were observed in 2% of patients (paralysis, paresis, paresthesia of the lower extremities, pneumothorax, pleural empyema) [2, 4, 14, 16, 21]. Intraoperative abdominal or thoracoscopic celiac plexus destruction by direct alcohol injection or surgical transection of ganglia have been applied with an efficacy comparable to the percutaneous technique [3].

The ideal procedure should preferably be highly efficacious, with low complication rates, and the least invasive. Proper visualization of the celiac plexus followed by precise administration of proper pharmacological agents all appear to be fundamental prerequisites for successful and safe neurolysis. Alcohol ablation is approximately twice as effective, compared with ablation using phenol [19]. Moreover, alcohol ablation is not associated with mutagenesis [19]. The introduction of EUS in the 1980s for imaging abdominal organs, including the pancreas, made it possible to precisely visualize the celiac plexus. The application of interventional endoscopy in the 1990s permitted the performance of controlled biopsies, drainage, or injection of drugs into tissues surrounding the stomach or duodenum under ultrasonographic guidance. Such procedures were previously performed surgically or percutaneously only.

Wiersema was pioneered celiac plexus neurolysis under EUS guidance in 1996, demonstrating high efficacy in patients with advanced abdominal malignancy (significant pain reduction in 79–88%) with low morbidity [7]. Subsequent studies confirmed these findings, showing a short-term success rate of 78%, which decreased to 30% after 12 weeks of follow-up, in particular, when no chemotherapy was applied [11].

In our study, the effectiveness observed early following treatment appeared lower, since significant pain reduction was reported in 69% of patients, while 31% had slight or no improvement. Late response to treatment, assessed 2-3 months following the procedure, was significant in a relatively large (56%) number of patients. 

The inability to completely control the pain in all patients as well as reduction of pain relief over time was observed in several studies [5, 8–10, 13, 20]. The reason why alcohol injection into the plexus did not completely eliminate pain may be explained by pathologic studies of the plexus following treatment [19]. Alcohol injection resulted in only partial destruction, and degeneration and fibrosis of nerve fibers and ganglia [19]. As a result, continued transmission of pain stimuli is still possible, although reduced in most patients [19]. The site of injection is also important and should preferably be performed at the most complex, ganglia-rich location within the celiac plexus. Bilateral injection (on both sides of the plexus) is more effective compared with single injection in the center of the plexus [17]. In order to destroy as many nerves and ganglia as possible, we routinely applied the triple injection method to the center of the plexus and bilaterally. This is different, compared with other reports that describe a single injection of a standard dose (20 mL) of the drug [11, 12, 16]. The prospective studies comparing different injection methods and applying different types of needles have a potential to explain differences in effectiveness of this procedure. In view of its limited efficacy, any efforts towards its improvement appear justified, including increasing the dosage and changing the mode of injection. Repeated procedures may also be of value in some cases, as is injection of steroids into the plexus in patients with chronic pancreatitis.

 Optimal timing for EUS-guided celiac plexus neurolysis is controversial. As in our study, it may be applied in a very advanced stage in patients requiring narcotic analgesia. Some authors, however, recommend the performance of neurolysis early in the course of disease, before pharmacotherapy with opioids has even been started [1, 16, 17]. In such cases, efficacy and safety may be increased.

 Our study shows that EUS-guided celiac plexus neurolysis is associated with a very low risk of complications. There was no significant treatment-related morbidity observed in any patients.

## 5. Conclusion

 EUS-guided celiac plexus neurolysis is a safe and effective treatment of severe pain in patients with advanced pancreatic cancer. It provides significant short-term pain relief in the majority of patients. However, its efficacy is limited, indicating the need for further studies aimed at improving the method.
